# Edge Computing Resource Allocation for Dynamic Networks: The DRUID-NET Vision and Perspective

**DOI:** 10.3390/s20082191

**Published:** 2020-04-13

**Authors:** Dimitrios Dechouniotis, Nikolaos Athanasopoulos, Aris Leivadeas, Nathalie Mitton, Raphael Jungers, Symeon Papavassiliou

**Affiliations:** 1 School of Electrical and Computer Engineering, National Technical University of Athens—NTUA, 15780 Zografou, Greece; papavass@mail.ntua.gr; 2 School of Electronics, Electrical Engineering and Computer Science, Queen’s University of Belfast—QUB, Belfast BT7 1NN, UK; n.athanasopoulos@qub.ac.uk; 3 École de Technologie Supérieure (ÉTS Montreal) | Université du Québec, Montreal, QC H3C 1K3, Canada; Aris.Leivadeas@etsmtl.ca; 4 Inria Lille-Nord Europe, Lille, 59650 Villeneuve d’Ascq, France; nathalie.mitton@inria.fr; 5 Institute of Information and Communication Technologies, Electronics and Applied Mathematics, Université Catholique de Louvain—UCLouvain, 1348 Louvain-la-Neuve, Belgium; raphael.jungers@uclouvain.be

**Keywords:** edge computing, internet of things, mobile robots, resource allocation, control co-design

## Abstract

The potential offered by the abundance of sensors, actuators, and communications in the Internet of Things (IoT) era is hindered by the limited computational capacity of local nodes. Several key challenges should be addressed to optimally and jointly exploit the network, computing, and storage resources, guaranteeing at the same time feasibility for time-critical and mission-critical tasks. We propose the DRUID-NET framework to take upon these challenges by dynamically distributing resources when the demand is rapidly varying. It includes analytic dynamical modeling of the resources, offered workload, and networking environment, incorporating phenomena typically met in wireless communications and mobile edge computing, together with new estimators of time-varying profiles. Building on this framework, we aim to develop novel resource allocation mechanisms that explicitly include service differentiation and context-awareness, being capable of guaranteeing well-defined Quality of Service (QoS) metrics. DRUID-NET goes beyond the state of the art in the design of control algorithms by incorporating resource allocation mechanisms to the decision strategy itself. To achieve these breakthroughs, we combine tools from Automata and Graph theory, Machine Learning, Modern Control Theory, and Network Theory. DRUID-NET constitutes the first truly holistic, multidisciplinary approach that extends recent, albeit fragmented results from all aforementioned fields, thus bridging the gap between efforts of different communities.

## 1. Introduction

The Internet of Things (IoT) consists of low-cost efficient sensors, actuators, and computing units and provides great benefits to people to synthesize a system of interrelated computing, sensing, and communication devices that facilitates and improves everyday life, in cities and industry. IoT is foreseen to reach 500 billion devices that are connected to the Internet by 2030 [1], while the global mobile traffic is expected to increase sevenfold by 2021 [2]. Though significant improvements have been obtained in terms of hardware advances and processing capabilities at the device level; still, in most cases, IoT devices (e.g., smart devices, sensors, actuators, mobile agents) cannot meet, and more importantly cannot guarantee the required high performance and/or fulfillment of time constraints, for time-critical and mission-critical IoT-enabled applications. Thus, offloading computation and energy intensive tasks to powerful computing infrastructure for further processing becomes of vital importance.

The success of the computation offloading, and consequently the performance of IoT-enabled applications, depends on many contextual parameters, e.g., the user’s mobility, various wireless parameters, and the resource availability of the computing resources in the data center. Most of the modern IoT-enabled applications rely on continuously moving people or mobile agents. Regarding the latter, various types of autonomous mobile agents or unmanned vehicles are used. Typical examples of these agents are the unmanned aerial vehicles (UAV), which are widely used in several human activities in the context of smart city, agriculture, area surveillance, rescue missions, and event coverage [3]. UAVs can be used individually or in a swarm, and they are equipped with various sensors in order to complete a mission or to execute their own tasks, such as trajectory planning and positioning. Their limited computing resources and energy reserves do not allow local data processing. Thus, the data offloading seems the only viable solution for using massively UAVs in various daily scenarios. Data are transmitted through wireless links, i.e., cellular or WiFi, and the quality of the wireless connection heavily depends on signal strength, interference, packet dropouts, and other parameters related to the wireless environment, which must be considered in the offloading decision.

The computation offloading aims to save time and energy at the end user’s side. Cloud computing seems the natural selection for offloading, as it is the prevalent service delivery model nowadays. However, the high network delay for sending data over public internet counterbalances the benefits of the powerful computing resources that are available at a cloud data center. Accordingly, Multi-Access Edge Computing (MEC) [4] and Fog Computing [5] have arisen as promising approaches to overcome this obstacle and provide the benefits of cloud computing in the proximity of the end-users. Over the last few years, powerful UAVs have been considered as a means to provide computing support to the end-users by acting as UAV-mounted MEC servers [6]. In that respect, the UAV-mounted MEC servers in combination with ground MEC servers collectively create a fog computing system [7], supporting end-users’ applications’ task offloading. Similarly, the use of clusters of UAV-mounted MEC servers is suggested [8], allowing the opportunistic task offloading to the neighboring UAV clusters with sufficient computing resources. In such a UAV-assisted network, computing intensive tasks are offloaded and executed in a nearby small-size edge data center, either directly connected with a wireless access point, or it is embedded on the UAV itself. The key difference between cloud and edge data center is that the latter has a finite amount of computing resources, which requires fine-grained resource management towards meeting the strict constraints of the deployed time- and mission-critical applications.

### The DRUID-NET Perspective and Contributions

This article presents the vision and perspective of the DRUID-NET (eDge computing ResoUrce allocatIon for Dynamic NETworks) framework, along with a detailed description of its main concepts and objectives. While considering the end-user’s mobility and the parameters of the wireless connection environment, the DRUID-NET framework aims at developing workload profile holistic and modular dynamic performance models of IoT-enabled applications based on the appropriate theoretical tools. Furthermore, the article aims to outline the control principles of novel resource management systems for these kinds of applications. In particular, the key research threads and topics of this article are summarized as follows:

- **Workload Profile**: The IoT applications generate time-varying traffic in terms of request size and number of flows. Additionally, the involved wireless communication between the mobile devices and the back-end software components introduces additional uncertainties that considerably affect the offloading decision and the resource scheduling at the edge computing infrastructure. Contrarily to existing average traffic characteristics, building dynamic traffic profiles and prediction mechanisms will enable more accurate, adaptive, and successful data offloading and resource allocation mechanisms.
- **Performance Modelling**: Most of the existing models for describing the performance of IoT-enabled applications are empirical and usually focus on a specific performance metric, e.g., response time, throughput, energy consumption, etc. These models cannot adequately capture the dynamic nature of the emerging applications, which in turn leads to either performance degradation or resource over-provisioning. On the other hand, the DRUID-NET framework proposes formal dynamic multi-input multi-output performance models applicable to various IoT applications. These types of models enable the design of novel controllers for finer regulation of Quality of Service (QoS) metrics.
- **Resource Allocation**: Usually, most studies in the literature combine a static performance model with solving an optimization problem. However, this approach assumes that the workload does not vary significantly, which limits their validity, applicability, and exploitability. In contrast to these approaches, we envisage to design stabilizing controllers in order to guarantee the feasibility of the resource scheduling and the performance requirements.
- **Control co-design** The DRUID-NET framework examines specifically the case where the application is the controller design and its implementation for dynamic processes. In this setting, the performance of the closed-loop system is considered in the overall application performance. The control co-design approach aims to design feedback mechanisms achieving closed-loop system properties such as reachability, stabilization, and other complex specifications, and simultaneously design and implement resource allocation algorithms for the dynamic network.

The rest of the article is organized as follows. In Section 2, the current state of the art is presented. Section 3 demonstrates the conceptual architecture of the DRUID-NET framework, while Section 4 describes three IoT-enabled use cases where the proposed solution is applicable. Finally, Section 5 draws the conclusions and future directives of our research.

## 2. Related Work and Motivation

This section provides a thorough yet comprehensive presentation of the most relative studies to the DRUID-NET framework, in the recent literature. Aligned with the DRUID-NET objectives, Abdelzaher et al. [9] presented five challenges on IoT applications and Edge Computing. This study focused mostly on deep learning-based application modeling, optimal offloading, closed loop guarantees, and collaborative offloading. Towards these directions, the related work is categorized under three major classes; (i) IoT workload profile, (ii) performance modeling and resource allocation, and (iii) control co-design.

### 2.1. IoT Workload Profile

The estimation of the workload and communication patterns in IoT-Fog/Edge networks has only been explored a little due the high heterogeneity of co-existing devices. Nevertheless, there is no doubt that the proper estimation of the offered workload and communication patterns could lead to a more efficient utilization of the underlying infrastructure.

Authors in [10] considered a two-tier network architecture consisting of shallow and deep cloudlets, and explored the benefits of hierarchical capacity provisioning based on queuing analysis. Although shown to be efficient in very specific cases, this approach cannot be generalized in principle. Osmotic Computing [11] relied on the deployment of lightweight microservices on resource-constrained IoT platforms at the network edge, coupled with more complex microservices running on large-scale datacenters. MobiQoR [12] introduced a new metric, Quality of Results, to validate the quality of edge resource deployment. Nevertheless, none of these approaches attempted to estimate the IoT workload, which in turn could significantly enhance the corresponding deployments. The authors of [13] and subsequently of [14] analyzed the resource allocation of a three-layer infrastructure (IoT, Edge, Cloud) under dynamic network conditions. However, they took into consideration the dynamic opt-in and out of IoT devices into the network, while ignoring their instantaneous workload generation. To the best of our knowledge, the only attempts to estimate workload are referring to the cloud utilization [15,16], and as such they did not capture the locality of the heterogeneous IoT traffics.

A promising approach to derive workload profile is to use machine learning techniques. Applying deep or machine learning techniques for IoT applications is not new [17], but most of the time they are centralized and do not need any adaptation to fit specific IoT devices limitations. In DRUID-NET, we will rely on existing estimation methods, such as [18], to estimate the workload of hardware constrained devices. In existing works, the focus is placed on one specific resource each time (e.g., energy, memory, computing, etc.) [19]. The DRUID-NET framework aims at extending them to multiple resources, while combining these approaches with predictive methods, which have only been slightly explored for IoT due to resource limitations. Thus far, methods such as ARIMA [20], deadreckonning [21], Kalman filters [22], Thompson sampling [23], or Bayesian approaches [24] have mainly been investigated for navigation and position prediction [20], data reduction [24], link prediction [25], or medium occupation [23]. Our aim is to provide a unique distributed and adaptive multi-resource estimation and prediction suitable for IoT devices. The DRUID-NET goal is to derive some communication patterns, clearly defined in time and size, towards assessing the need in edge resources in time and space. This edge-resource sizing combined with performance modeling, controlled mobility of edge-resource and resource allocation, will enable the adaptive deployment of sufficient resources, on demand, and in an efficient manner.

### 2.2. Performance Modeling and Resource Allocation in Cloud and Edge Computing

Resource allocation has become one of the most important open research problems in Cloud and Edge computing and IoT. In the cloud computing environment, the computing resources are assumed to be infinite; thus, static or empirical models combined with coarse resource scheduling techniques have been shown sufficient to provide high performance through over-provisioning. However, these approaches are neither optimal nor able to provide QoS guarantees. Regarding application’s performance modeling, the empirical or fixed models considered already known request sizes and execution times, which are not only hardware-specific, but generally very difficult to be precisely computed. Furthermore, many studies relied on queuing models [26], e.g., G/G/1 or G/G/n, which are reliable only for steady state. It is obvious that this kind of modeling cannot capture transient phenomena due to dynamic workload demand. With this capacity, System Theory [27] can provide dynamic modeling methodologies, appropriate for Cloud/IoT-based applications. The interesting reader may refer to survey [28] for an extended analysis of control theoretic approaches on performance modeling and cloud elasticity. Close to DRUID-NET concepts, Dechouniotis et al. [29] proposed Linear Parameter Varying (LPV) modeling of cloud applications combined with set-theoretic controllers to guarantee a feasible solution of the elasticity in cloud data centers, while Leontiou et al. [30] derived fuzzy Takaki–Sugeno models and designed robust controllers to address simultaneously the problems of vertical and horizontal scaling, and load balancing with stability guarantee.

Contrarily to cloud computing, the resources of edge computing are rather limited; thus, static allocation techniques cannot achieve optimal resource utilization. Furthermore, modern time- and mission-critical IoT-enabled applications [31,32] have strict performance requirements that only dynamic modeling and intelligent allocation algorithms can guarantee. Similarly to cloud, in the edge computing context, most of the relative studies proposed static models alongside with the optimization of a single performance criterion, e.g., energy consumption or response time. Towards this direction, Sonmez et al. [33] proposed a two-stage fuzzy mechanism for offloading requests to edge and cloud infrastructure. The set of fuzzy rules are empirically decided and the VM (Virtual Machine) utilization modeling is threshold-based, which is applicable only for specific types of IoT applications. Queec [34] formulated the problem of scheduling multi-user tasks to multiple edge nodes as an optimization problem which minimizes the overall offloading latency of all tasks. Jalali et al. [35] analyzed fixed flow-based and time-based energy consumption models, and they presented a detailed comparison on energy consumption between cloud and edge computing systems under various network settings. Lyu et al. [36] presented a collaborative Cloud-MEC-IoT architecture and proposed a request modeling scheme and an admission control framework to address the scalability problem of these platforms. Although the authors considered heterogeneous edge resources, the computation model was not dynamic. The authors of [37] addressed both the problems of network selection and service placement for MEC infrastructure. Towards the reduction of the complexity of the general problem, they decomposed it into a series of sub-problems and solved them in an iterative fashion. However, the proposed performance model focused only on network related parameters ignoring the processing time of the application.

In the 5G era, Network Functions Virtualization (NFV) and Software Defined Networks (SDN) play key roles for the realization of many type of verticals, which are comprised of several IoT applications. In this context, virtualized and isolated Service Chains (SCs) comprised of a series of Virtualized Network Functions (VNFs) implemented as VMs need to be deployed in the available MEC infrastructure to offer networking services to the IoT traffic. Normally, the objective of this kind of resource allocation mechanism aims to minimize the overall deployment cost (e.g., the computational and communication resources that an SC needs in order to be provisioned) [38]. Another common approach is to minimize the overall delay, since several IoT applications are characterized as mission critical and delay sensitive. Thus, a valid approach is to utilize the MEC resources that are closer to the IoT devices [39]. An alternative approach to minimize the delay is to create resource clusters inside the MEC infrastructure, where the various requested SCs can be deployed [40]. Minimizing the number of clusters and appropriately positioning the VNFs can lead to a reduction of the communication delay. Efforts have also been dedicated to optimize the energy consumption. The authors of [41] modeled the energy dissipation of the resources in the IoT and MEC infrastructures and constructed a Linear Programming algorithm to carefully select the resources to place the SCs. Another objective focuses on the optimal allocation and scheduling of the available edge resources. This objective can be translated into either: (a) minimizing the overall resource usage, to enable multiple heterogeneous SCs, servicing heterogeneous IoT applications, to co-exist in the MEC layer [42], or (b) minimizing the resource idleness of the infrastructure [43]. Load balancing can also be applied by minimizing the maximum link utilization and reducing the bandwidth consumption [44]. This can be achieved by adopting appropriate queuing and QoS modeling during the optimization problem to minimize the resource utilization [45]. Even though all the above solutions target valid and open challenges of resource allocation in the IoT/MEC, they only propose static approaches failing to provide a holistic mechanism that takes into consideration a multi-objective and dynamic solution. Following the performance modeling and control design principles of [9,46], DRUID-NET aspires to provide multi-variable dynamic models and design modern control methodologies that ensure the desired user’s performance requirements and optimize the utilization objectives of the infrastructure provider simultaneously.

### 2.3. Control-Theoretic Resource Allocation and Control Co-Design

In control theory, the effect of a shared, imperfect communication network between the controller and the sensor/actuator network has been studied extensively for almost three decades, generating the separate branch of Networked Control Systems (NCS), Ref. [47,48]. NCS suffer from many non-idealities. For instance, networked induced delays or, even worse, packet dropouts occur, as the information from the sensor to the controller or from the controller to actuator(s) can be lost in a time interval. Moreover, due to the limited energy available at decentralized nodes, bandwidth can be low, so that the effect of quantization in the communication channels may not be neglected. In addition, switching or hybrid phenomena may occur due to the asynchrony between disconnected agents, or due to event-triggered strategies. Finally, the computational problem, to be performed at the nodes, may be part of a global optimization problem, which is split into decentralized subtasks.

Several methods have addressed these non-idealities separately. Time delays, for instance, have been tackled utilizing perturbation theory, Lyapunov stability theory, and hybrid systems analysis, but also probabilistic methods involving Markov chains and stochastic automata [49]. Quantization problems have led to a rich literature, where the controllability of a plant subject to quantized control is ruled by the so-called *entropy* of the system [50]. From the hybrid control point of view, researchers from real-time computing have dealt with the schedulability problem of distributed control settings, leading to the design of several protocols for a stable closed-loop behavior, [51,52,53]. Decentralised computation/optimization has been another major topic of research in Systems and Control [54]. Here, though the state of the art is rich, the interaction of this constraint with others is not well understood and studied. Let us note however that the consensus problem has been deeply studied, in many settings, e.g., quantized communications [55].

Additionally to the stochastic results [56], recent theoretical work on the controllability and observability properties of the NCS [57] has shown that a more refined modeling of the communication network allows the proper definition and verification of such properties, thus adding new tools to the NCS community. Furthermore, proof-of-concept work has shown that, under a new modeling framework for hybrid systems and specifically constrained switching systems [58], the control performance can be directly associated with the network quality [59].

Rather than designing the control and communication protocol in two steps, co-design methods aim to synthesize simultaneously controllers and the communication patterns (sampling, delays, scheduling protocols). Applied only to networked control systems with constrained communication resources so far, co-design methods have been extensively studied the last decade [60,61,62,63]. Perhaps the most relevant breakthrough in this area is the emergence of event-triggered and self-triggered control mechanisms that allow asynchronous sampling, thus reducing the network traffic, while at the same time behaving sub-optimally [64,65,66].

Nevertheless, there is limited work on the co-design of controllers taking into account simultaneously more than one phenomena (schedulability, network utilization, edge resource utilization, energy consumption, etc.) caused by the distribution of computing and communication resources. It is anticipated that the research developments in the upcoming decades will allow for encapsulating, comparing, and subsequently altering the impact of the several non-idealities, and this in turn will have a significant impact on future control applications, where resources must be used parsimoniously, in balance with the constraints and the overall considered objective. This will require and motivate new paradigms in Systems and Controls, where multi-objective optimization, model-free (data-driven) approaches, approximate optimality (however with firm safety guarantees), reconfigurability, and resilience take a central place.

## 3. DRUID-NET Conceptual Architecture

**Figure 1:**
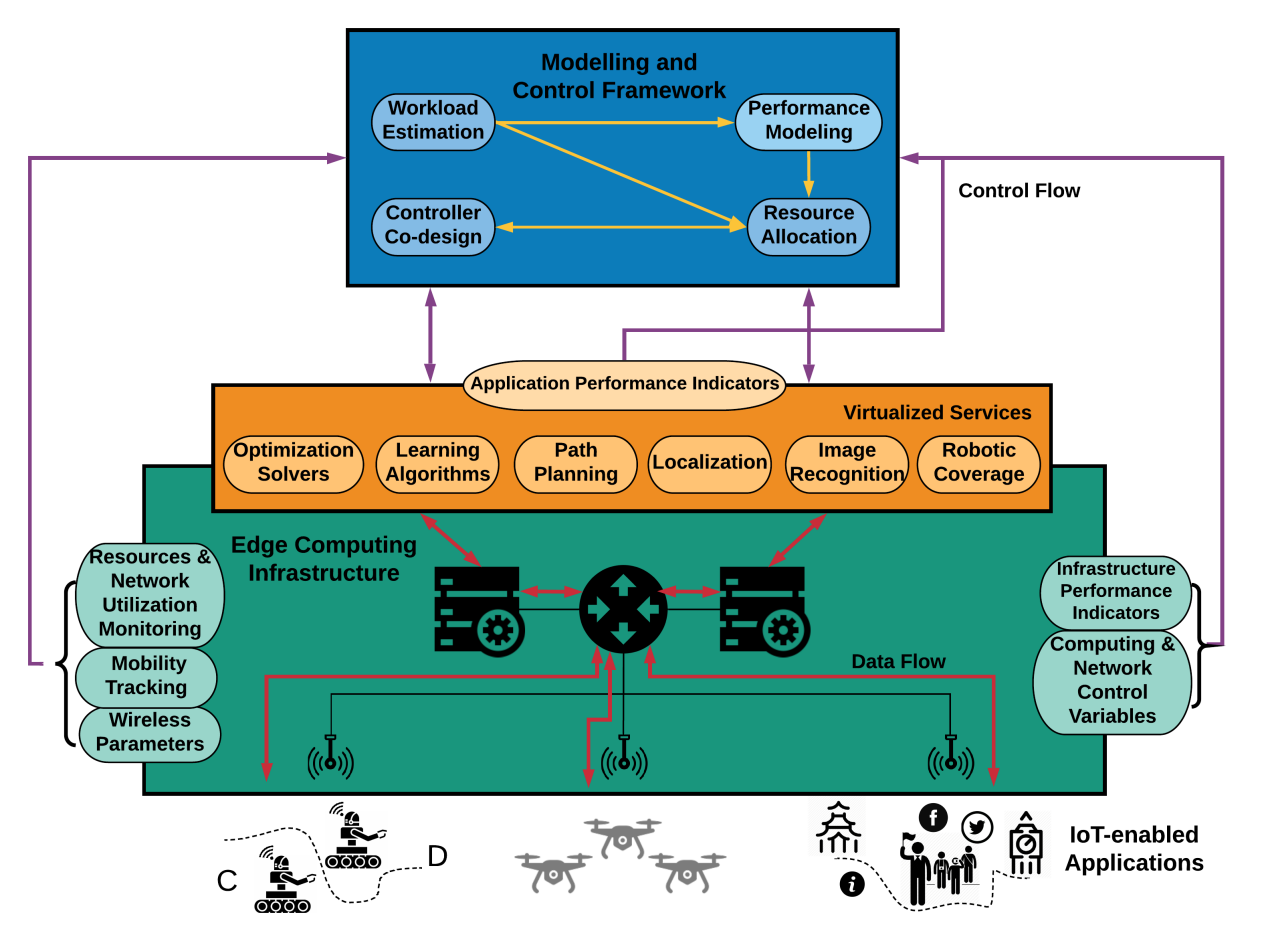
Conceptual architecture.

Figure 1 illustrates a high-level overview of the overall DRUID-NET framework. The architecture follows the NFV/SDN paradigm and separates the flow of information into control and data planes. At the lowest layer, the IoT applications are deployed, and the generated workload (data flow) can be offloaded for further processing at the upper level of Edge Computing. In this layer, any component of the application is provided as a virtualized service. As it is shown in the figure, a virtualized service corresponds either to IoT specific functionalities, e.g., path planning and image recognition, or control components such as learning algorithms or optimization solvers. The modeling and control framework collects information (control flow) about the status of the computing and network infrastructure at the edge computing level in order to create workload-resource profiles, update the performance model for every application, and realize the feedback control mechanism for the resource allocation, while simultaneously implementing a resource-aware control strategy for the cyber-physical system to be controlled (control flow). This holistic approach allows the application’s dynamical modeling taking various contextual information into account. Furthermore, the controller co-design treats the resource allocation algorithms as application components in the virtualized services. Each major component of the modeling and control framework is described in more detail in the following subsections.

### 3.1. IoT Workload Profiling

As mentioned before, a major challenge for solving the resource allocation problem in edge computing settings is to predict the time-varying characteristics of the workload/traffic, as different traffic flows and volatile conditions can influence significantly the resource allocation mechanism. Aspects such as the load generated from an IoT device, latency specifications, the transmitting data frequency, the wireless protocol, the mobility of the devices, and the number of devices associated with the IoT gateway, change the amount of resources requested from the edge, while also influencing the scheduling process. Until now, only generic traffic models have been proposed to estimate the traffic aggregated at the edge layer, while stationary IoT devices are assumed, leading to a static rate model, which however limits its effectiveness and applicability in real scenarios.

Going a step beyond from the pertinent literature, which only considers average and general traffic characteristics of the IoT applications (e.g., Brownian motion as one-fits-all model), DRUID-NET framework aims to differentiate and categorize the requirements of various IoT applications using appropriate data analytic and mathematical models. In particular, we classify and categorize the IoT applications by leveraging the transmission patterns, the spatial and temporal correlation of the traffic, as well as other traffic related characteristics such as the frame size distribution, and the burstiness of the traffic of the IoT applications. The novelty of this approach is that we create prediction mechanisms to treat the dynamics and uncertainty in the corresponding traffic profiles. Each predictive mechanism targets specific categories of IoT applications with similar requirements and characteristics to define the type, the size, as well as the time and the location of the requested resources. Furthermore, with this approach, we can dispose the erroneous assumption that specific tasks are associated with static and pre-specified resource footprints. In contrast, we replace this analogy with an opportunistic association between the requested resources and the IoT traffic dynamicity, thus introducing a holistic mechanism inspired by data analytics, and traffic analysis methods.

#### 3.1.1. IoT Applications Classification

A first classification of the IoT applications can be produced by simply answering yes or no, to questions regarding the involved “things”/devices. Indicative such questions can be identified as follows: (i) Are the devices heterogeneous? (ii) Are they battery-powered? (iii) Are they sending data with high or low frequency? (iv) Are they data rich (e.g., multiple number of sensor measurements)? (v) Are the devices mobile?

The answer to such questions will help us to create a first clustering of the IoT applications. These clusters will contain IoT applications with similar device characteristics and behavior. Nonetheless, this first-phase categorization does not necessarily mean that the IoT applications belonging in the same cluster will present the same exactly resource requirements at the Edge. The reason is that different network access technologies can significantly affect the network requirements of the IoT applications. For example, different access technologies (e.g., LoRaWAN, Wi-Fi, IEEE 802.15.4, cellular, etc.) have different characteristics in terms of packet length, transmission range supported, MAC mechanisms, topological characteristics of the associated IoT devices (e.g., star, mesh, peer-to-peer), number of device connections supported, etc.

Thus, DRUID-NET takes into consideration both the functional and network requirements of the IoT applications in order to provide a complete and realistic IoT application classification.

#### 3.1.2. IoT Applications Workload Prediction

The above categorization will help us to extract the workload generated from each cluster of applications in terms of bandwidth, latency, and other important Key performance Indicators (KPIs) during the offloading of IoT tasks to the Edge. Specifically, through this approach, we can propose appropriate mathematical models to simulate the traffic behavior of the various IoT applications. Nonetheless, even with this modeling, a lot of ambiguity will exist. The reason is that IoT access networks include several uncertainties, usually being wireless, lossy, and unreliable. Hence, the goal of DRUID-NET is not only to categorize and classify IoT applications based on their traffic profiling, but to also apply network analytics to make the communication as deterministic as possible.

Our goal is to replace the so far average estimates of the IoT applications with instantaneous and accurate transmission metrics. To this end, appropriate machine learning algorithms (i.e., Thompson, ARMA, Bayesian) need to be integrated in the traffic profiling in order to learn and predict the network conditions between the IoT and the Edge. This can be decisive in the performance of the subsequent resource allocation at the Edge. The Edge controller will be able to adapt to and predict the changing workload arriving at the Edge infrastructure, creating a holistic and realistic resource allocation approach.

### 3.2. Performance Modeling

The available resource models are usually single-input single-output. Energy or response time are typically the model’s outputs, while computing resources (e.g., CPU, memory), incoming requests, and network bandwidth are the control variables. In most of the current studies, the relation between input and output is fixed and empirically derived. For example, the processing time of a request is proportional to its file size and inversely proportional of the service rate measured in CPU cycles or millions of instructions per second. Although this assumption is reasonable, the actual processing time depends on several time-varying parameters, which are not easily measured. Furthermore, in combination with static resource allocation mechanisms, the offloading decision performs adequately only for specific operating conditions, being unable to guarantee stability under fluctuating workload and heterogeneous IoT communication infrastructure.

Contrary to current approaches that provide empirical static models, we aim to develop formal, realistic, and dynamic traffic and resource models applicable to emulate the generated traffic from various IoT applications. For this purpose, DRUID-NET adopts hybrid dynamical models [67] that have the capacity to include several performance metrics (i.e., state variables) and resources as control parameters (input variables). This type of modeling takes into account in a single formulation the various contributions of the diverse objectives and constraints to the performance/cost. This framework moreover allows for discovering the trade-offs between accuracy, complexity of representation, and real-time feasibility of the resource allocation strategy. Furthermore, the chosen framework will be capable of capturing structural changes interpreted as discrete jumps in the dynamics, e.g., user mobility, change in wireless protocols and topology, and addition/removal of edge servers. Finally, alongside with the dynamic models, the DRUID-NET framework aims to identify the uncertainties of these models and quantify their boundaries in order to facilitate the design of the respective control laws.

### 3.3. Resource Allocation

The workload profile estimator and the dynamic model of the resources and the overall status of the network/servers provide the foundation upon which the resource allocation algorithm will be developed. Specifically, the objective is to develop a joint communication, computing and storing virtualization paradigm that is updated and adapted dynamically. For this purpose, we consider the problem of simultaneously (i) allocating storage, computing and communication resources, (ii) modifying network topology/ protocol, and (iii) structuring the edge computing data centres (such as VM distribution). Two distinct approaches relating to static and dynamic resource allocation are considered.

#### 3.3.1. Static Resource Allocation

In this approach, we do not take into account the dynamic nature of the processes under study; however, we consider the full resource allocation problem. The method is oriented towards solving multi-objective optimization problems fast that will in turn provide the optimal operating point for the communication network, and the computing and storage allocation in the edge/cloud servers. Our goal is to describe the complex interrelations between the aforementioned resources in an analytic manner, merging available models, e.g., from queuing theory and Markov models. Next, we plan to solve the optimization problems using mixed-integer, linear, and nonlinear programming. Since the complexity of these problems does not allow often exact real-time solutions, our intention is to propose approximate solution algorithms that provide guarantees of the level of suboptimality of the identified solution. Additionally, we will employ machine learning algorithms to relax the complexity of these highly nonlinear/nonconvex problems so they can be solved in real-time, thus respecting hard time constraints. This approach will focus on problems involving complex specifications and mostly static models, aiming to maximize the QoS delivered.

#### 3.3.2. Dynamic Resource Allocation

In this approach, the DRUID-NET framework proposes dynamic control-theoretic resource allocation mechanisms. Utilizing the models established by capturing the performance metrics dynamics in a hybrid dynamical system, our goal is to follow control-theoretic approaches that provide formal guarantees on important properties describing the resource allocation problem. For example, a main objective is to provide guarantees of the speed of convergence of the performance metrics to a pre-determined range, defined by translating the QoS requirements to mathematical statements. Moreover, our goal is to provide decision mechanisms that allow structural changes in some cases (for example, turning on and off edge servers in a cluster, changing the topology in a communication network), together with continuous strategies (such as CPU and memory utilization in a server). The natural, main challenge in this approach is the scalability of the decision algorithm, which will be tackled by proposing smart allocation strategies that allow trade-offs between performance and real-time implementation. Another challenge is to establish resource allocation mechanisms using only partial information, which is the most realistic scenario. This issue will be addressed by proposing distributed control mechanisms that take continuously into account local information and receive only intermittently information about the states of the whole system.

### 3.4. Co-Design of Controllers

As we have mentioned before, in the broad field of Systems and Control, several different paradigms have emerged in the last few decades, to deal with the control of IoT-enabled cyber-physical systems. Indicative examples include hybrid behavior, quantized control, varying delays, safety-criticality, nonlinear control, etc. Although these challenges are typically met together in IoT environments, the research activities have led to disconnected communities, and likewise very specific and custom control techniques that limit their implementability in a holistic framework. In a real-life IoT control application, these non-idealities take place all together. We argue that the different paradigms separately introduced for each of these non-idealities are hard to reconcile, thus the DRUID-NET framework is devoted to deploying the theoretical results in actual applications.

Modern IoT applications need controllers that address a mixture of these undesired phenomena. Our goal is to establish a formal decision mechanism that will be able to change the provisioning of the resources in real time, adapt its control objective to the available bandwidth, weigh the cost of communication with respect to the advantage of involving decentralized agents, and eventually address a multitude of practical challenges appearing in networked, resource-constrained control applications. Such a new generation of controllers will be made possible by the merging of two sets of hybrid models, namely (a) the performance model having as internal variables performance metrics of the infrastructure and as inputs the resource distribution and utilization, and (b) the process model (having, for example, variables related to position, orientation, velocity and acceleration of mobile agents, lighting conditions, room temperature, mode of operation of sensors, etc). To provide an example of the challenges that will be met in this setting, let us raise the following question: what do traditional data-rate theorems from quantized control (see, e.g., [68]) become in an environment with packet losses and varying delays, and varying computational resources? Another important line of research that will be necessary to follow in a time-varying resources setting is to categorize and model the complexity of the control algorithms. Allowing their dynamic adjustment will eventually provide the coupling between the process/application to be controlled and the control algorithm resource provisioning. In turn, this will enable (i) the establishment of real-time control mechanisms with formal guarantees for the closed-loop system, and (ii) the optimal utilization of resources, either in the network or the edge.

## 4. IoT-Enabled Applications

The proposed architecture is generic enough offering a holistic paradigm, while its estimation, modeling, and control methodologies are applicable in several categories of IoT applications, such as the ones based on mobile agents (e.g., UAVs) or designed for crowded smart areas or emergency scenarios. The following subsections demonstrate three representative use cases of the DRUID-NET framework.

### 4.1. Collaborative Robotics

**Figure 2:**
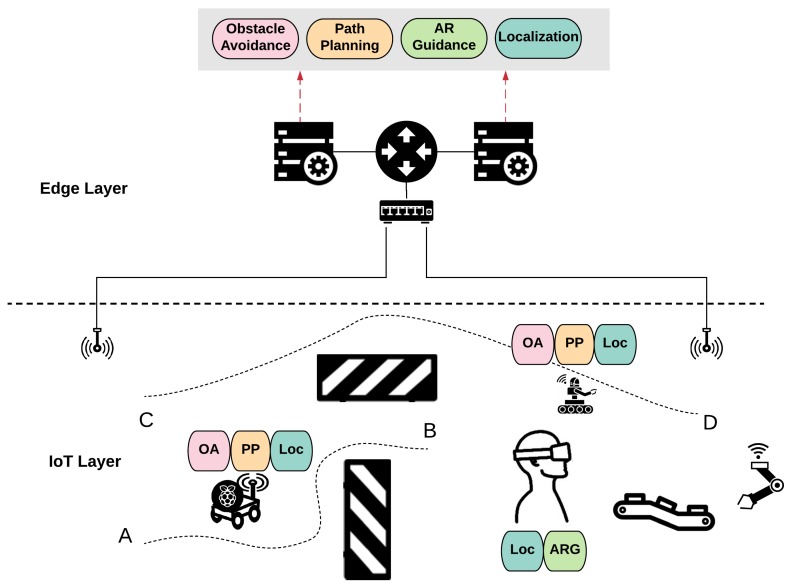
Human–Robot collaboration.

Collaborative robotics is a prerequisite for Industry 4.0, especially in the Industrial Internet of Things setting. The current trend is to produce and program robots that have the capability to work together, or in close proximity, to humans in a shared environment. Removing a physical (or virtual) cage from the robot brings many challenges, the most critical of which is guaranteeing safety/avoiding collision, without leading to an unsatisfactory performance, e.g., the robot working in a non-acceptable speed. The setting can be extended to the case where there are many robotic agents and humans sharing the factory floor, or any other indoor or outdoor environment, e.g., a logistics warehouse, an airport, swarm UAVs networks, etc. In all aforementioned cases, similar challenges appear, namely: (i) intermittent and noisy measurements of the position of the agents either by static sensors or sensors mounted on the robots, (ii) faulty wireless communication networks, (iii) stringent safety specifications as humans and robots move freely in the same environment, and (iv) time-critical specifications. These challenges become harder when the computing/storing/communication resources are limited, or not always available to a control application, which is the typical case. Thus far, a few approaches, aligned with the ones appearing in the cyber-physical systems control problems, take explicitly into account a part of these challenges, e.g., [69]. Controllers, which are co-designed with the resource allocation and computation offloading mechanisms, can be used for human–robot collaboration in the IoT-enabled environment or for real-time, large scale coordination of mobile robots. It should be noted that an additional control challenge in this case, additional to the presence of constraints, is the complex temporal specifications that need to be satisfied. Currently, the control objective has moved away from just ensuring stability or tracking for a prespecified set of reference trajectories, to satisfying statements, for example “robot A and B should collaborate towards a task X and eventually return to their initial positions if these positions are not occupied”, as shown in Figure 2. These control applications are often time-critical as well as safety-critical; thus, a very careful co-design procedure should be developed for the controller that leads to formal guarantees without requiring many, possibly idle, resources.

This scenario enables, and is enabled by, a combination of almost all components of the DRUID-NET framework, namely workload and resource estimation, and control co-design of a set of control applications in a platform where resources are shared and their availability is volatile.

### 4.2. Rapid Resource Deployment for Physical Disaster Scenarios

In the case of a physical disaster, the fixed communication infrastructure could be destroyed or unavailable due to high workload demand. Furthermore, for rescue operations, it could be critical to deploy additional on-demand computing and network resources at the proper place and time, in order to alleviate any remaining network infrastructure and collect data from remaining communicating devices such as mobile phones or sensors, towards helping to locate and rescue survivors. Mobile agents, especially UAVs, are suitable for these kinds of missions and can provide additional edge resources capable of processing the data at low latency and organizing the rescue operation. In order to serve the survivors devices as much as possible, there is a need to predict the kind and amount of resources these devices will request and the location of these resources. Some UAV-mounted edge resources may need to be deployed sporadically and temporarily at different locations based on IoT devices needs and mobility. Thus, there is a need to anticipate the deployment of edge services and to estimate the time they will be required at a given place to decide whether it is worth deploying durable edge resources, or instead mobile temporary resources could suffice. In this latter case, the estimation of the location and quantity of required resources should be anticipated to allow their timely deployment. The deployment of edge resources will be such that a maximum of IoT devices can be served within the required latency, either directly or through multi-hop communications. Direct communications will be favored for devices with very-low latency requirements, while multi-hop communications could be used for weaker latency requirements non-necessary communications. The trajectory of distributed UAV should be consciously planned accordingly, taking into consideration the time restrictions (robots should be deployed at the proper place before we need them).

**Figure 3:**
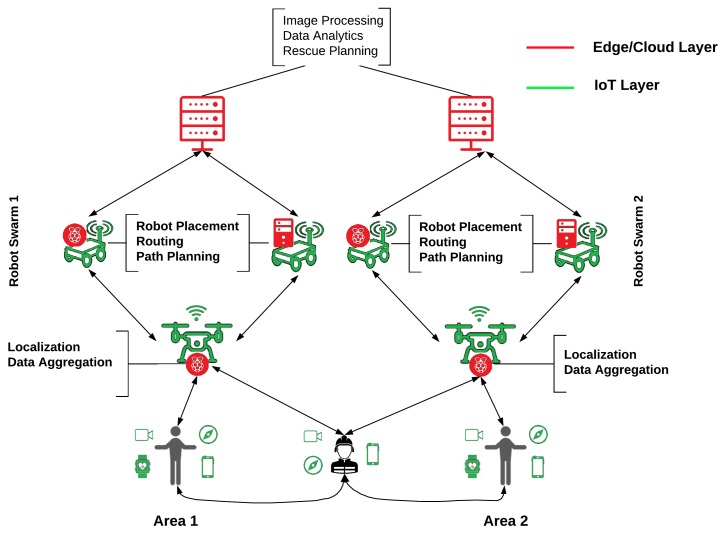
Rapid resource development for physical disasters.

Figure 3 illustrates the operation of the proposed framework under a physical disaster scenario, such as for example the occurrence of a gas leakage in a large factory. In this case, swarms of mobile robots will be deployed in order to find victims or survivors that require immediate medical assistance. Two types of mobile robots can be deployed, namely, (i) Unmanned ground vehicles (UGVs) and (ii) UAVs. UGVs will cover the ground area (x,y dimensions), while UaVs can provide a certain altitude coverage (z dimension) or coverage in non accessible areas by the UGVs (e.g., upper floors, atriums, etc.). Normally, we expect to find more obstacles in the ground area (e.g., offices, machines, shelfs), which can be translated in a higher number of UGVs in comparison with the UAVs (a ratio of 2:1), as shown in Figure 3. The goal of the interconnected UAVs is to locate living or dead persons, while at the same time send footage of the interior of the factory in order to create a 3D visualization of the area. In this manner, the users of the application (e.g., fire brigade) can immediately detect persons in need and send help to the corresponding location, eliminating the risk of long exposure to harmful gas for the rescuers. For the path planning of the mobile agents, the robots will be capable of detecting the Wi-Fi or LTE preamble and accordingly plan the route towards the source of the signal. The notion behind this behavior is that normally people have in close vicinity their mobile phones or other wireless devices (e.g., smart watches). This will facilitate the path planning and the pointless roaming of the UAVs in space. In order to prioritize the traffic and eliminate the impact of poor wireless communication, the swarm of robots can intensify the load of images/video and increase their quality only in areas with high probability of detecting a person, and send this traffic at the Edge for further processing. UGVs can approach the victims and sense if they are living or unconscious (e.g., detect eye movement, detect sound, etc.). When a UGV finds a survivor, it can communicate with the UAV of the swarm nearby, which in turn can lower its altitude close to the position of the person in need and drop an oxygen mask until help arrives.

In this case, using swarms of mobile robots will assist in eliminating the non-essential communications. Combining service differentiation and smart data offloading to UAVs, there will be reduction of any unnecessary communication between users and overhead due to extensive signaling. Since the number of available robots may still be inadequate to serve all ground services, the prioritization of the applications, flows, and devices is of paramount importance for the success of critical missions. Under these circumstances, the priority will be given to the areas with many victims or of major importance for the completion of the mission; thus, the available swarms of mobile robots should be distributed accordingly. Even in the case of homogeneous UGVs and UAVs with identical computing and networking capabilities, the optimal allocation of UAVs or UGVs formulates a dynamic optimization problem, which depends on the size of the damaged area, the communication ranges of both UGVs and UAVs, the flying altitude of UAVs, the propagation conditions, the data communication requirements (amount of data, frequency of collection, etc.) and the number and type of devices to serve. For example, if the victims are equally spread in different locations, the robot swarms would be equally scattered to deploy their resources in these areas so that the maximum number of devices would be served directly. On the other hand, the mobile robots will be driven to the most damaged area in order to serve the required network traffic.

This scenario illustrates the use and combination of the different control components of the DRUID-NET framework, and in particular: (1) workload estimation in quantity, time, and space, (2) resource allocation (tasks assignments to UAV and/or robots) and (3) path trajectory.

### 4.3. Mobility-Aware Edge Computing

**Figure 4:**
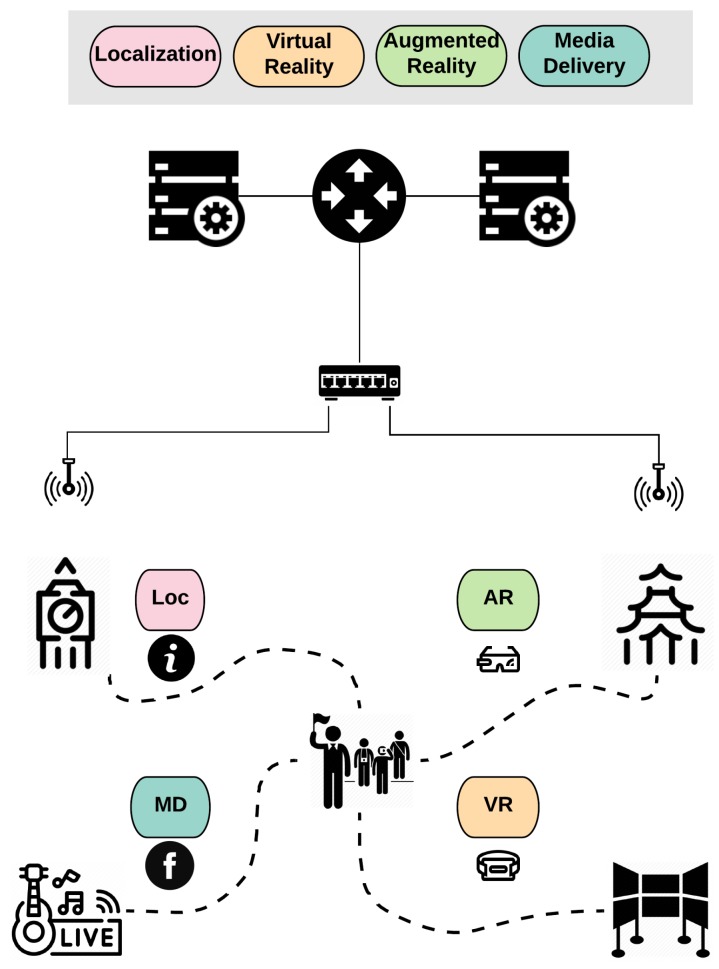
Mobility-aware edge computing.

Most of the modern smart city applications rely on mobile end-devices of continuously moving humans. Thus, the user’s mobility is a dominant parameter of IoT systems. As shown in Figure 4, in the case of an urban touristic areas, e.g., museums and squares, the visitors collect information about Points of Interests (PoIs) (i.e., exhibits or social events) using their mobile devices. For example, leveraging the augmented or virtual reality technologies, they can retrieve media-enriched information about the surrounding PoIs. However, it is prohibited for the mobile end-devices with limited resources to run these types of applications locally. Thus, the edge computing infrastructure is essential to host the smart applications and meet the user’s QoS requirements. Additionally, in crowded touristic areas, the number of visitors varies significantly during short-term (i.e., a day) or long-term periods (summer or winter); therefore, an accurate prediction methodology is important for optimal resource scheduling. Moreover, the offloading decision should be based on both the user’s transmission capability and the availability of edge resources. With this capacity, in order to maximize the admittance of users, the main features of the overall generated traffic should be extracted alongside with patterns of the user’s mobility. Then, utilizing the dynamic models, effective controllers can be designed towards the horizontal and vertical scaling of resources and the simultaneous guarantee of any QoS and Quality of Experiment (QoE) requirements.

This use case illustrates the necessity and the collaboration of the involved components of the DRUID-NET framework. Particularly, workload estimation, dynamic performance modeling, and resource allocation components interact to meet the respective requirements and optimize the resource utilization under varying workload conditions.

## 5. Conclusions

This article presents the most important challenges of IoT-enabled applications, along with the perspective and the basic concepts and objectives of the novel DRUID-NET framework. The corresponding components of the DRUID-NET framework are carefully designed to address several emerging challenges at any level of the IoT/Edge/Cloud system, stemming from mobile end-devices up to powerful cloud data servers.

In particular, the workload estimation aims to create a profile of IoT applications that includes features of the generated data, parameters of the wireless connection, and patterns of the user’s mobility. The performance modeling components identify the multi-input multi-output dynamical systems that capture the dynamic operation of the applications, and are utilized to design the resource allocation and the offloading decision strategies. The resource allocation component is in turn responsible for deciding any control action at any level of the hierarchical system. Depending on the objectives of the controller, the resource allocation can be either static or dynamic, providing guarantees on QoS metrics, e.g., response time or energy consumption, and system properties, such as stability. Finally, the co-design of the controllers enables the binding between the IoT application and the resource control algorithm in order to provide guarantees for the closed-loop system.

The DRUID-NET framework aspires to verify its modular architecture and components through different IoT scenarios. These use cases are carefully selected in order to cover all main challenging and emerging aspects of the IoT applications and Edge computing paradigm.

## Abbreviations

The following abbreviations are used in this manuscript:

MDPI	Multidisciplinary Digital Publishing Institute
DOAJ	Directory of Open Access Journals
IoT	Internet of Things
UAV	Unmanned Aerial Vehicle
UGV	Unmanned Ground Vehicle
MEC	Multi-Access Edge Computing
DRUID-NET	eDge computing ResoUrce allocatIon for Dynamic NETworks
QoS	Quality of Service
QoE	Quality of Experience
NFV	Network Functions Virtualization
SDN	Software Defined Networks
5G	Fifth Generation
SC	Service Chain
VNF	Virtualized Network Function
VM	Virtual Machine
NCS	Networked Control Systems
